# Supplementary material for: A Conserved Acidic Residue in the C-Terminal Flexible Loop of HIV-1 Nef Contributes to the Activity of SERINC5 and CD4 Downregulation
Source: Viruses. 2023 Feb 28;15(3):652. doi: 10.3390/v15030652 (PMC10057511; doi:10.3390/v15030652)
Supplement: Supplementary file 1 [file viruses-15-00652-s001.zip › viruses-2161392-supplementary.pdf]

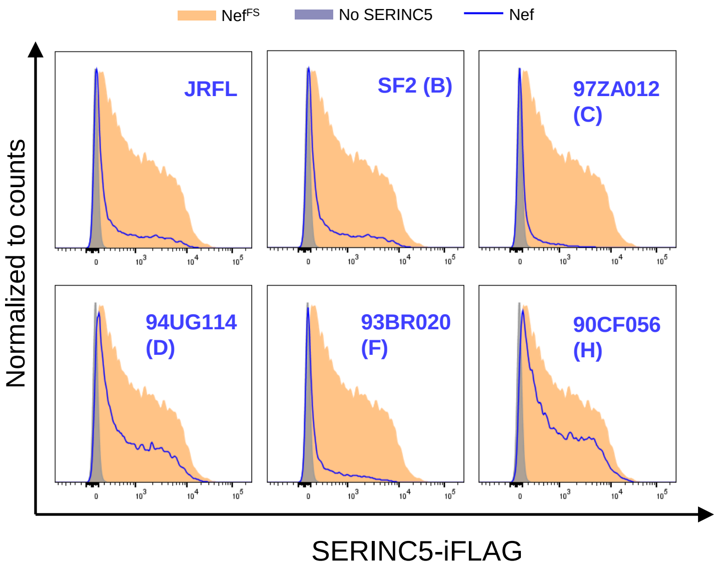

Figure S1
FACS plots measuring SERINC5-iFLAG cell surface levels in cells co-transfected with the indicated *nef* alleles expressed upstream of an IRES2-eGFP cassette to allow exclusive gating of Nef-expressing cells.

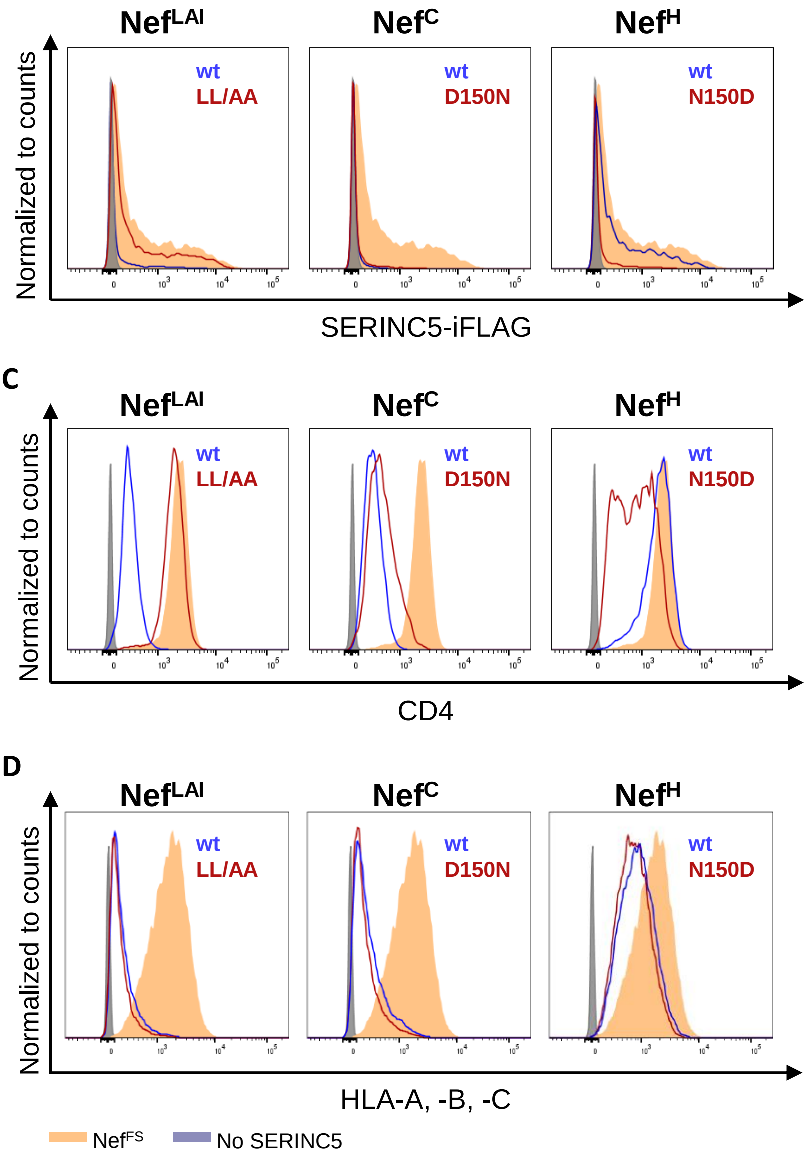

Figure S2

FACS plots measuring SERINC5-iFLAG **(a)**, CD4 **(b)** and MHC-I **(c)** cell surface levels in cells expressing the indicated Nef molecules expressed upstream of an IRES2-eGFP cassette to allow exclusive gating of Nef-expressing cells. The well-characterized mutation of the di-leucine sorting signal in the context of the Nef^{LAI} protein was also tested as control.

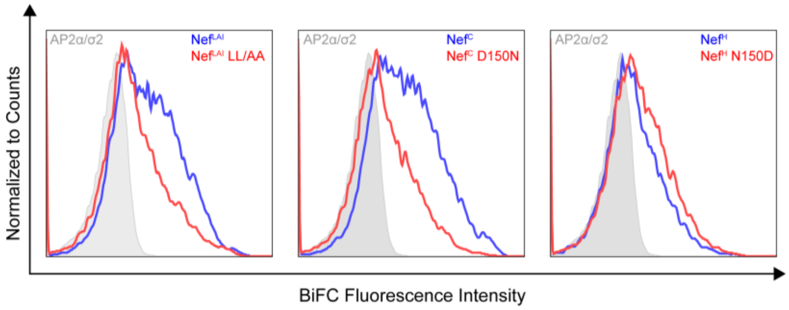

Figure S3
FACs plots measuring mean fluorescence intensity of BiFC signal in cells expressing indicated Nef proteins tagged with Venus N-HA and AP2α/σ2 hemicomplex tagged with V5-Venus C-terminal fragment.
